# Research progress of anti‐γ‐aminobutyric acid B receptor encephalitis and a case report of paraneoplastic associated encephalitis and treatment analysis

**DOI:** 10.1002/ibra.12017

**Published:** 2022-02-10

**Authors:** Yi‐Kun Lv, Hai‐Qing Zhang, Jun Zhang

**Affiliations:** ^1^ Department of Neurology Affiliated Hospital of Zunyi Medical University Zunyi China

**Keywords:** anti‐γ‐aminobutyric acid B receptor encephalitis, epilepsy, peripheral lung cancer

## Abstract

Encephalitis is one of the common diseases in neurology. Early diagnosis and appropriate treatments are essential. Autoimmune encephalitis (AE) generally refers to a type of encephalitis mediated by autoimmune mechanisms. It is gradually considered to be an important cause of reversible encephalitis caused by noninfectious factors. It can occur in children, adolescents, and adults, and is clinically characterized by multifocal or diffuse brain damage such as personality changes, seizures, and cognitive impairment, with an overall good effect of immunotherapy. According to the clinical features of the patients, blood and cerebrospinal fluid tests, neuroelectrophysiology, cranial imaging, treatment and prognosis, AEs can be broadly divided into specific antigen (antibody)‐related AEs and nonspecific antigen (or antibody) ‐related AEs. With the development of AEs research, more and more anti‐neuron antibodies have been found, which provides an important reference for the diagnosis and treatment of AEs. Understanding the knowledge about AEs is important to discover new diseases and deepen the understanding of the immunopathological mechanisms of existing central nervous system diseases. Anti‐γ‐aminobutyric acid B (GABA‐B) receptor encephalitis is a type of AE, but this disease is rare in AE, often develop to the clinical manifestations of marginal encephalitis, accompanied by obvious seizures or status epilepticus, Some patients had tumors, mainly small‐cell carcinoma, prompt diagnosis, early immunotherapy and, if necessary, tumor treatment resulted in complete or partial neurological improvement in most patients.

## Graphical abstract

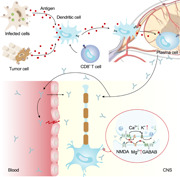

## 1 INTRODUCTION

In recent years, autoimmune encephalitis (AE) has been gradually recognized by clinical practitioners, and more and more treatment options are being used in clinical work due to the discovery of types that were difficult to detect in the past. GABA‐B receptor encephalitis accounts for approximately 5% of all cases of AE.^1^ In this paper, we review the progress of clinical studies related to GABA‐B receptor encephalitis to facilitate early diagnosis and treatment. We also provide a case of GABA‐B receptor encephalitis from onset, diagnosis and treatment to clinical prognosis, and observe the efficacy of this patient, which is only a reference for clinicians in the treatment of this disease.

## 2 CASE INFORMATION

The patient, male, 54 years old, was admitted to hospital with “episodic loss of consciousness with twitching of the limbs for 4 days and a recurrence of 5 h.” Four days ago, he had twitching of the limbs with no apparent cause and then became unconscious, unable to respond to calls, with closed teeth, eyes staring upwards, salivation, blue lips, recovered in about 3–5 min, complained of numbness of the limbs before the twitching. He was disoriented, no incontinence, no dizziness, no headache, no nausea, no vomiting, no cough, no sputum, no chest tightness, no shortness of breath, occasionally in a trance, left shoe on, right shoe on, no trousers on, and so on. He was found lying on the roadside 5 h before admission and woke up about 3–5 min later. After waking up, he had no memory of the events and did not recognize his relatives, so he consulted the emergency department of the author's hospital and was admitted to the neurology department with “cerebral infarction and epileptiform seizure” after a cranial computed tomography (CT). He was previously healthy. There was no specific personal history. No similar cases in the family. Neurological examination: confusion, unresponsiveness, decreased memory, calculation, and judgement. There was no restriction in the elevation of the soft palate, the uplift of the soft palate was not restricted, the uvula was in the centre, the pharyngeal reflex was present, the tongue extension was slightly leftward, no abnormality was found in other nervous system.

**Figure 1:**
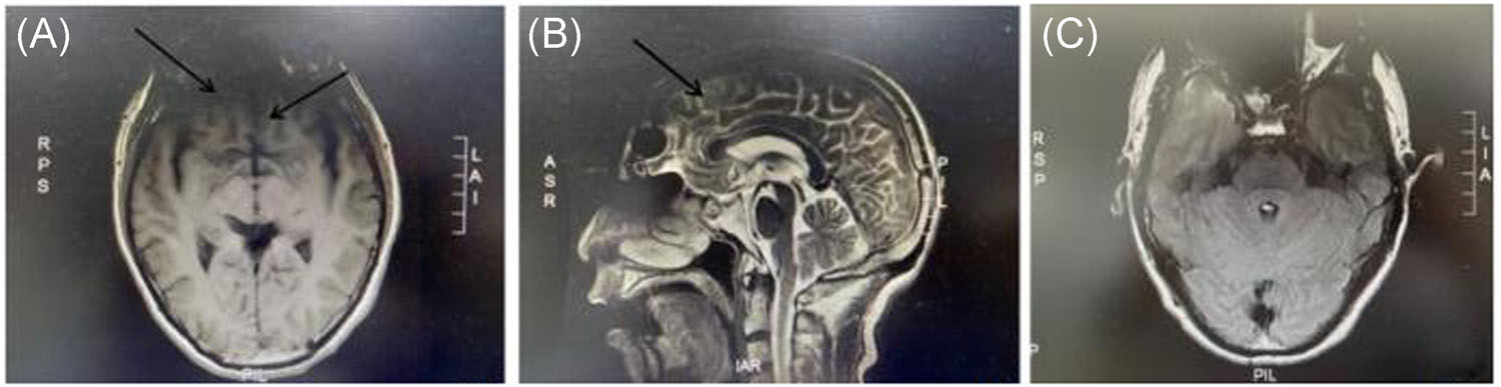
Cranial MRI examination. The sulcus near the knee of the corpus callosum widens (A, arrow), unclear display of some structures in frontal lobe (B, arrow), FLAIR image examination (C) showed no abnormal signal in hippocampus and amygdala. MRI, magnetic resonance imaging [Color figure can be viewed at wileyonlinelibrary.com]

**Figure 2:**
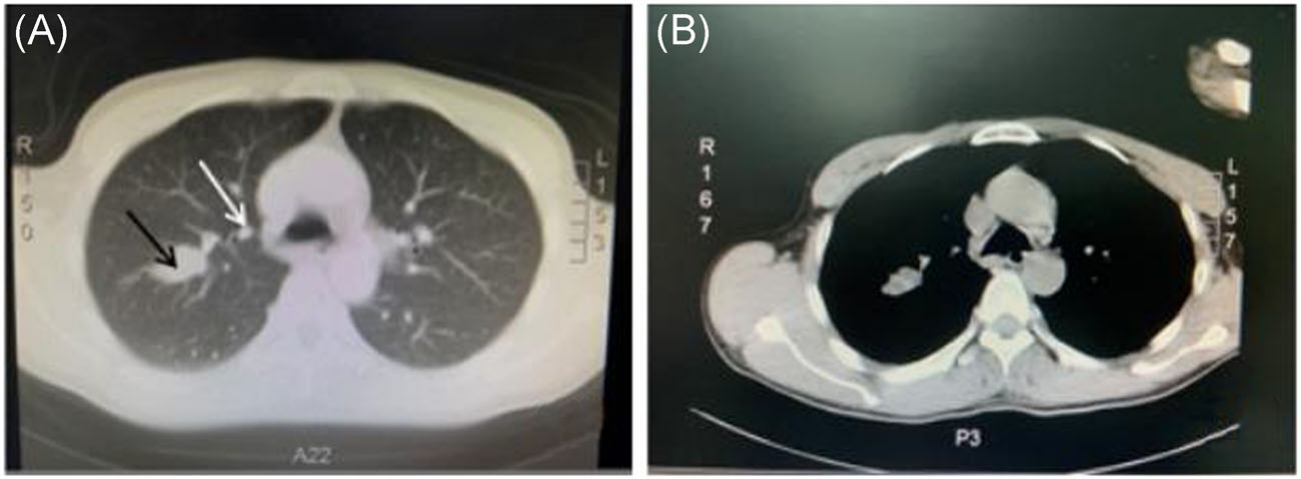
Chest CT examination. Irregular mass in the right upper lobe, about 27 mm × 18 mm (A, shown in black arrow), right hilar soft tissue mass (A, shown in white arrow), enhanced scan showed irregular soft tissue density shadow in the right hilar region, with uniform density on enhanced scan (B, shown in white arrow). CT, computed tomography [Color figure can be viewed at wileyonlinelibrary.com]

**Figure 3:**
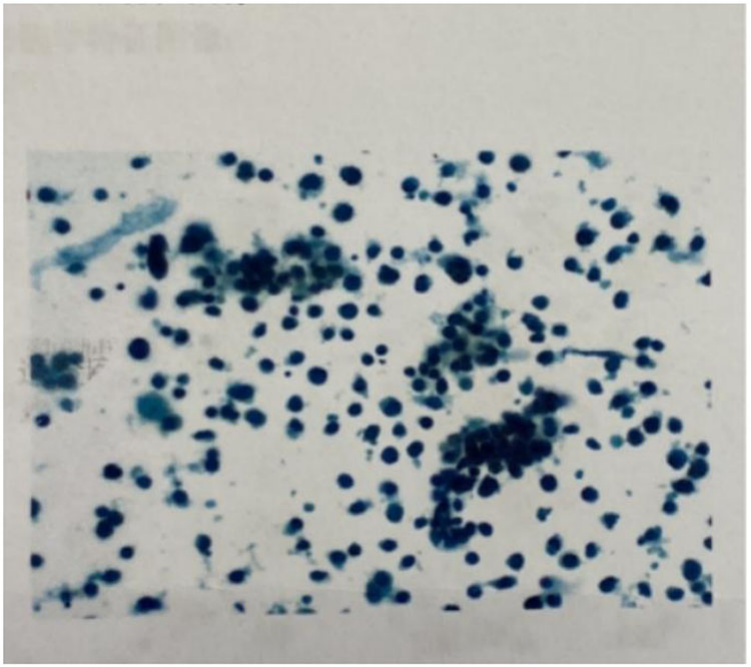
Cytopathology of lymphatic puncture in patients. (2R + 11RS lymph node) cytopathology showed more atypical small cells [Color figure can be viewed at wileyonlinelibrary.com]

**Figure 4:**
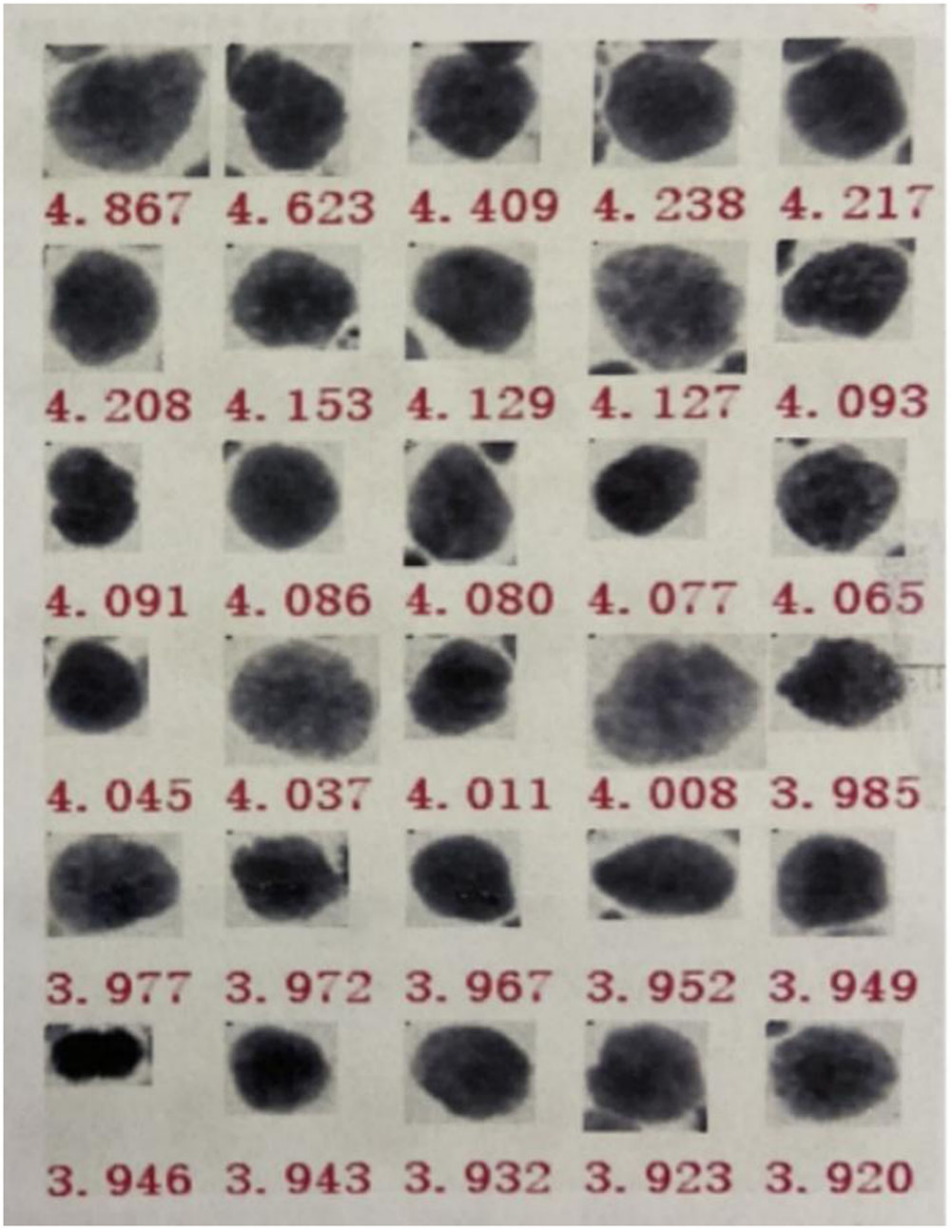
DNA ploidy of lymph node cells was detected. (2R + 11RS lymph node) punctured cells with abnormal DNA ploidy (≥3) [Color figure can be viewed at wileyonlinelibrary.com]

Auxiliary examination: magnetic resonance imaging (MRI) of the head and MRI of the hippocampus were performed: some structures in the frontal lobe were not visible bilaterally. There was no abnormality in the hippocampus bilaterally (Figure 1). CT‐enhanced scan of the chest: nodules in the upper lobe of the right lung, tending towards peripheral type Ca, soft tissue masses in the right hilar region and enlarged lymph nodes were considered (Figure 2). A lumbar puncture was performed and clear, transparent cerebrospinal fluid was seen, with a cerebral pressure of 140 mm H_2_O (1 mm H_2_O = 9.8 Pa). Cerebrospinal fluid routine: total cerebrospinal fluid cell count 110 × 106/L, white blood cell count 54 × 106/L, neutrophils 8%, lymphocytes 92%. Cerebrospinal fluid biochemistry: chloride 126.4 mmol/L, glucose 3.73 mmol/L, cerebrospinal fluid protein quantification: 408 mg/L (normal range: 200–400 mg/L). Pulmonary tumor‐associated antigen: squamous epithelial cell carcinoma antigen: 2.3 ng/ml. Dynamic video EEG monitoring showed a small amount of low‐amplitude 15–25 Hz β‐wave activity recorded in all leads of the brain and more low‐amplitude δ‐wave activity in both hemispheres. Serum AE profile: anti‐GABA B receptor antibody lgG++ 1:100. BLOT method: anti‐Amphiphysin antibody lgG (+), all others negative. Cerebrospinal fluid AE profile: anti‐GABA B receptor antibody lgG++ 1:10. And bone ECT: increased bone metabolism in the axillary segment of the 6th–7th rib on the right side, with a high possibility of malignant lesions. The lung cancer with lymph node metastasis in the right hilar and mediastinal 4R regions; (1) the lesion in the axillary segment of the right 7th rib was not metabolically high and was considered benign; (2) multiple small lymph nodes in the IB and II regions of both necks, bilateral axillae and bilateral groins were shown. Bronchoscopic tissue strips and cytosol were sent for cytopathological + DNA ploidy analysis: (2R + 11RS lymph nodes) heterotypic cells were found, which did not exclude small cell carcinoma (lung) (Figure 3) cellular DNA ploidy test results: abnormal DNA ploidy cells (≥3) were seen (Figure 4).

The diagnosis of paraneoplastic‐associated AE was confirmed by a comprehensive analysis of the condition.

After admission, the patient was given sodium phenobarbital 0.1 g intramuscularly Q12h, diazepam 10 mg intravenously Q12h and sodium valproate 500 mg orally Bid to control the seizures, and the number of convulsions decreased significantly. He was transferred to our thoracic surgery department to prepare for surgery because of the family's strong desire for surgery. After transferring to the thoracic surgery department, the convulsions did not recur, but there were still psychiatric symptoms, and the family considered that surgery was risky, so he was transferred to the neurology department for further treatment. He was treated with brain support, acid suppression and stomach protection, anti‐infection, and protection of liver function. He was also given aripiprazole 5 mg oral Bid, olanzapine tablets 10 mg oral Qd, and oxcarbazepine tablets 0.3 g oral Bid antiepileptic treatment, as the patient had occasional psychiatric symptoms and could not be taken off immunosuppression, if surgical treatment was chosen, perioperative After further consideration of the risks involved, the family opted to admit the patient to the oncology department for chemotherapy using the EP regimen: etoposide 0.1 g IV infusion Qd, lopressor 50 ml IV infusion Qd, and symptomatic supportive therapy. At follow‐up the patient is stable, with occasional psychiatric symptoms, and is currently being treated with prednisolone tablets 5 mg oral Qd hormone therapy, and continued with oxcarbazepine tablets 0.3 g oral Bid for seizure control.

The patient is now seizure‐free, with tangential questions and answers, for complete memory loss in the last 2 years, and no abnormalities in the remaining higher neurological functions.

## 3 PATHOGENESIS

**Figure 5:**
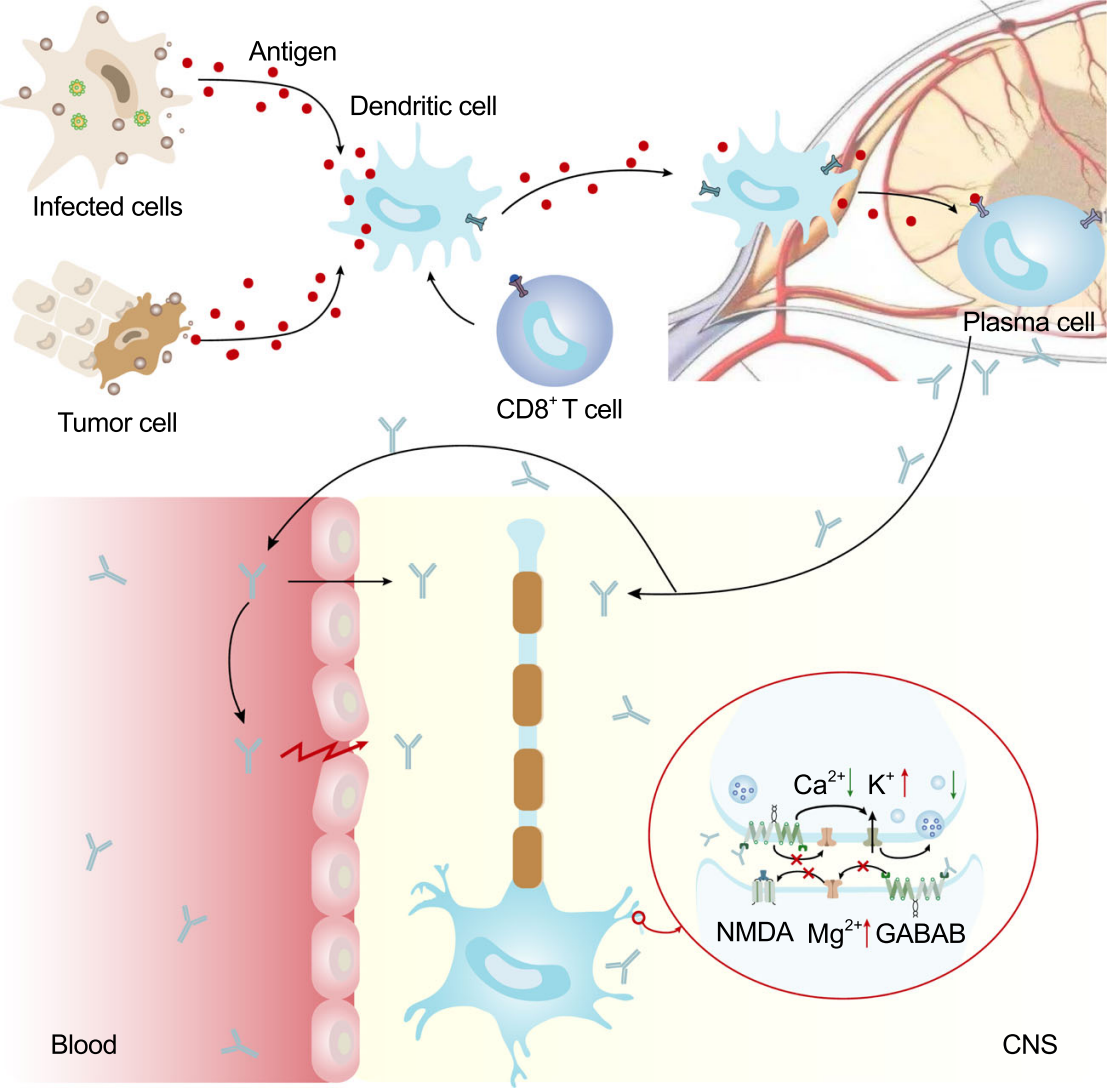
Pathogenesis of GABA‐B receptor Phalitis. Inhibitory nerve conduction, resulting in the inhibition of neuronal activity [Color figure can be viewed at wileyonlinelibrary.com]

GABA is the major inhibitory neurotransmitter in the central nervous system and plays a key role in regulating neuronal activity. GABAB receptors are widely distributed in the cerebral cortex, hippocampus, cerebellum, and thalamus, and are inhibitory synaptic proteins in neurons that play an important role in neurotransmitter transmission and synaptic plasticity. The metabotropic GABAB receptor is a G protein‐coupled receptor consisting of two subunits, GABAB1 and GABAB2. It is activated by G proteins and mediates slow and persistent inhibitory neurotransmission in the brain with the formation of chloride channels that promote potassium inward flow and calcium channel inhibition, leading to suppression of neuronal activity. GABAB receptors have important regulatory roles in many circuits of the nervous system and are associated with learning, memory, and cognitive functions, and their dysfunction may lead to epilepsy, cognitive impairment, and behavioral abnormalities. Golombeck et al.^2^ reported three cases of non‐paraneoplastic anti‐GABAB receptor limbic encephalitis and one case of paraneoplastic anti‐GABAB receptor limbic encephalitis associated with small cell lung cancer, with pathogenesis associated with the induction of autoantibody production and CD^8+^ T‐cell pathogenic effects from various causes (Figure 5).

## 4 EPIDEMIOLOGY

AE has been studied extensively by scholars at home and abroad, and has been classified by foreign researchers^3^ as paraneoplastic encephalitis, encephalitis associated with anti‐cell membrane surface antigen antibodies or anti‐prominent protein antibodies, and encephalitis associated with other systemic autoimmune diseases. AE is not a rare cause of encephalitis. However, its prevalence is not well defined. Since 2010, the introduction of autoantibody testing to certain medical facilities in China has led to an increase in the number of patients diagnosed with the disease each year. Four thousand one hundred and six patients with unexplained encephalitis were studied at Peking Union Medical College Hospital between May 2013 and December 2014, and this study showed that the incidence of AE had exceeded that of viral encephalitis, with 531 (12.9%) patients testing positive for autoantibodies and anti‐N‐methyl encephalitis. Of these, 531 (12.9%) patients tested positive for autoantibodies and 423 (10.3%) for anti‐*N*‐methyl‐d‐aspartate receptor (NMDAR) antibodies, suggesting that anti‐NMDAR encephalitis is the most common cause of AE.^4^

## 5 CLINICAL MANIFESTATION

Patients with GABAB receptor‐resistant encephalitis usually have a subacute onset, and the age of onset ranges from 18 to 85 years, with a prevalence in children aged 60–70 years, and also in children aged 3 years. Most of the clinical manifestations of GABAB receptor‐resistant encephalitis are similar to those of limbic encephalitis, with seizures, memory loss, psychiatric disorders, and impaired consciousness as the main manifestations.^5^, ^6^ The typical clinical presentation of patients with anti‐GABAB receptor encephalitis is usually epilepsy^7^, ^8^, ^9^ followed by autonomic disturbances, and some investigators have divided the clinical presentation into an epileptic phase and a subsequent encephalitic phase. Seizures are a prominent clinical feature in the early stages of anti‐GABAB receptor encephalitis and are usually not controlled by antiepileptic drug therapy alone, with approximately 64.3% developing persistent status epilepticus, which may be related to the immune response causing sclerosis of the hippocampus.^10^ All patients present with an encephalitis phase, which manifests as diffuse brain damage including cognitive and behavioral impairment and seizures. The encephalitis phase often presents early with confusion and focal or generalized seizure continuity, and about 1/3 of patients develop autonomic disorder episodes with central apnea and bradycardia, which may be life‐threatening. After the encephalitis phase, severe paracrine amnesia and disorientation remain the main features of the patient, which may be related to pathological changes in hippocampal neurons, with a slow and progressive recovery of symptoms.

## 6 CLINICAL FEATURES

Blood and cerebrospinal fluid antibody tests remain one of the mainstays of diagnosis. Anti‐GABAB receptors can be detected in the serum, cerebrospinal fluid, or both in patients with anti‐GABAB receptor encephalitis. The clinical manifestations are titer‐dependent, and changes in antibody titers are closely related to the clinical course of the disease.^11^ High titers are associated with encephalitis, whereas low titers are associated with seizures, rigid receptor syndrome, and oculoclonus myoclonus.^12^

Anti‐GABAB receptor titers are tumor‐related and usually range from 1:10 to 1:240. Patients with tumors have high antibody titers, up to 50 times that of nontumor patients, suggesting that clinical testing of antibody titers can predict the likelihood of a patient having a tumor. However, antibody titers do not correlate with disease severity. Anti‐GABAB receptors may coexist with a variety of other autoantibodies. A case report of a patient with AE with positive anti‐IgLON5 antibodies in serum and cerebrospinal fluid was accompanied by positive anti‐GABAB receptor antibodies in serum, but the patient's main clinical symptoms were determined by the positive antibodies detected in the cerebrospinal fluid.

Patients with anti‐GABAB receptor encephalitis often lack specificity on cranial MRI,^13^ and some patients may have normal imaging despite their disease. Although the patient's imaging presentation may be variable, the ability to find the characteristic features of AE in the limbic structures can alert the clinician to a timely diagnosis and therapeutic measures. Patients often show extensive T2‐FLAIR high‐signal lesions outside the limbic system, in addition to the classic MRI imaging presentation.^14^ Anti‐GABAB receptor encephalitis mainly presents with multifocal unilateral or bilateral subcortical MRI T2/FLAIR signal abnormalities, usually involving the temporal lobe in 95% of cases. These T2/FLAIR changes may disappear during the course of the disease or appear as new lesions and rarely show contrast enhancement, with a more limited correlation with patient symptoms. These MRI findings are important because they are common not only in anti‐GABAB receptor encephalitis but also rarely in other AE, which provides valuable clues for clinical identification of anti‐GABAB receptor encephalitis. Most electroencephalography (EEG) records only have nonspecific characteristics^7^, ^14^ (Table 1).

**Table 1:** Clinical features of GABA‐B receptor encephalitis

Anti‐GABAB receptors	Cranial MRI	Electroencephalogram
High titers: encephalitis	T2: Extensive hyperintense lesions	Nonspecific characteristics
Low titers: seizures, rigid receptor syndrome and myoclonus	T2/Flair: Abnormal signal, usually involving the temporal lobe	
Anti‐GABAB receptor titers range from 1:10 to 1:240: tumors		
Antibody titers were 50 times higher in cancer patients than in non‐cancer patients		

Abbreviation: MRI, magnetic resonance imaging.

## 7 TREATMENT

For the time being, the main treatment methods of the disease include immunotherapy and tumor resection. The patients with primary tumor are mainly treated with tumor therapy, while the treatment of nontumor patients is mainly treated with clinical immune regulation or immunosuppressive therapy. Studies have shown that 60%–70% of patients in the acute phase of anti‐γ‐aminobutyric acid B receptor encephalitis can achieve partial or complete remission after immunotherapy. First‐line treatment mainly includes glucocorticoids, gamma globulin, and plasma exchange. Second‐line treatment mainly includes rituximab, cyclophosphamide, and so forth. For patients who are insensitive to first‐line treatment, second‐line drugs may be considered. In patients with tumors, treatment with antitumor is able to significantly improve the prognosis.^15^, ^16^ Clinical work suggests that immunotherapy should not be used alone for seizure control, but in combination with antiepileptic drugs to facilitate for seizure control and that oral immunosuppression may maintain antiepileptic drugs at the lowest effective dose to reduce side effects.^2^ With appropriate immunotherapy, patients with AE have a reduced seizure frequency and do not appear to require long‐term use of antiepileptic drugs for seizure control. Younger patients are more likely to achieve seizure freedom after discontinuation of antiepileptic drugs compared to adults. In patients with anti‐GABAB receptor encephalitis, antiepileptic drugs can be tapered once seizures are controlled, whereas in patients with post‐encephalitis brain injury and medial temporal lobe atrophy, long‐term antiepileptic drug use may be required for seizure control.^17^

## 8 DISCUSSION

An AE is a noninfectious, brain disorder associated with antibodies of the central nervous system. Among them, anti‐γ‐aminobutyric acid B receptor encephalitis is an AE with positive cell surface antigen (synaptic protein) antibody, with borderline encephalitis symptoms such as seizures, cognitive impairment, and psycho‐behavioral abnormalities as the main manifestations.^18^

GABA is the main inhibitory neurotransmitter in the central nervous system and produces postsynaptic inhibitory effects mainly by activating G protein‐acting ion channels, including limiting the duration of neural network excitation and preventing excessive neuronal synchronization.^19^, ^20^ It is widely distributed in the brain and spinal cord, with the highest levels of distribution in the cerebral cortex, hippocampus, thalamus, and cerebellum.^17^ GABAB receptors, as inhibitory receptors, mediate presynaptic inhibition mainly by inhibiting potassium and calcium channels, but the pathogenesis is unknown.

The typical clinical manifestations of patients with anti‐γ‐aminobutyric acid B receptor encephalitis are seizures, cognitive dysfunction, altered consciousness, and mental status, of which 80% of patients have seizures as the first symptom,^21^ and atypical clinical manifestations are opsoclonus‐myoclonic syndrome and ataxia.^22^ The discovery of anti‐γ‐aminobutyric acid B receptor antibodies in cerebrospinal fluid and blood is a specific indicator for the diagnosis of the disease,^23^ while routine and biochemical examinations of cerebrospinal fluid are nonspecific, and most patients with anti‐GABAB receptor encephalitis have normal or mildly elevated cerebrospinal fluid pressure and white blood cell count, elevated protein, and mostly normal sugars and chlorides.^19^, ^21^ It may be helpful in the diagnosis of the disease. Approximately 66% of patients with anti‐gamma‐aminobutyric acid B receptor encephalitis typically have imaging findings of high signal intensity in the medial temporal lobe on brain MRI FLAIR sequences,^19^ or atrophy of the hippocampus and frontotemporal lobe.^24^ In this disease, it is currently reported that the majority of EEG can be characterized by epileptic discharges of temporal lobe origin, as well as diffuse or scattered slow waves,^21^ but no typical epileptiform discharges were observed in the EEG of this patient, and the EEG waveform was positively correlated with the severity of the patient's state of consciousness, but there was no significant specificity, and 24‐h ambulatory video‐EEG monitoring may help in the diagnosis, and whether there is research value for this disease needs to be further explored.

It has been reported in the literature that the proportion of this disease with tumors is about 50%, chest CT or PET suggests pulmonary malignant tumors, and the main tumor type is small cell lung cancer.^19^ In this case, chest CT examination performed in an outside hospital before admission revealed right lung occupying lesions, and relevant puncture cytopathology also supported the diagnosis of small cell lung cancer. Because the patient's psychiatric symptoms were poorly controlled in the early stage of the disease, the family chose chemotherapy, so no surgical plan was taken for tissue biopsy.

The main treatment methods of the disease include immunotherapy and tumor resection. First‐line treatment mainly includes glucocorticoids, gamma globulin, and plasma exchange. Second‐line treatment mainly includes rituximab, cyclophosphamide, and so forth. For patients who are insensitive to first‐line treatment, second‐line drugs may be considered. In patients with tumors, treatment with antitumor is able to significantly improve the prognosis. In this paper, the patient was admitted to the hospital to improve the relevant examinations, and began to use high‐dose methylprednisolone sodium succinate pulse followed by oral prednisolone tablets and other glucocorticoid pulse therapy. The condition improved significantly, which was consistent with the report.

Compared with other AE, anti‐γ‐aminobutyric acid B receptor encephalitis has a poor prognosis, mostly dies of lung cancer progression, and survives for about 1–2 years.^16^ Therefore, early diagnosis of this disease, detection of lung cancer, and early intervention have significantly improved the prognosis of patients with anti‐γ‐aminobutyric acid B receptor encephalitis.

In this paper, the patient could not stop hormones in the perioperative period because of long‐term recurrent psychiatric symptoms, and EP chemotherapy regimen was selected. It is worth pondering the contradiction that the long‐term use of immunosuppressive agents in the treatment of this disease affects surgical treatment.

In summary, AE has gradually become a common disease in clinical work, and there are few clinical cases of anti‐γ‐aminobutyric acid B receptor encephalitis. When patients are found to have prominent seizures with mental and behavioral abnormalities in clinical work, attention should be paid to screening. If conditions permit, cerebrospinal fluid and blood autoimmune encephalitis antibody tests are taken after admission to confirm the classification and diagnosis of AE. For patients with poor response to immunotherapy, it is necessary to consider whether they have tumors to carry out antitumor treatment as early as possible. The use of immunosuppressive agents and perioperative preparation in surgery require appropriate assessment of related risks and better and reasonable treatment options. If tumor resection cannot be performed, chemotherapy can be taken as early as possible. When the patient's condition is stable and can be weaned from the use of immunosuppressive agents, it is considered whether tumor resection can be selected. Only when tumor and AE are treated at the same time, certain clinical efficacy may be achieved.

## CONFLICT OF INTERESTS

The authors declare that there are no conflict of interests.

## ETHICS STATEMENT

There were three authors in the article, all of whom agreed to sign it and submit it for publication. There are no multiple submissions for this article. We have obtained informed consent from study participants. This article research work has no fund source.

## AUTHOR CONTRIBUTIONS

Yi‐Kun Lv contributed to the conception of the study; Jun Zhang helped perform the analysis with constructive discussions; Hai‐Qing Zhang revised and finalized it.

## TRANSPARENCY STATEMENT

The authors affirm that this manuscript is an honest, accurate, and transparent account of the study being reported; that no important aspects of the study have been omitted; and that any discrepancies from the study as planned (and, if relevant, registered) have been explained.

## Data Availability

The availability of the data concerning the case is related to the diagnosticexaminations that the patient submitted during hospitalization.

## References

[1] Lancaster E , Martinez‐Hernandez E , Dalmau J . Encephalitis and antibodies to synaptic and neuronal cell surface proteins. Neurology. 2011;77(2):179‐189. 10.1212/WNL.0b013e318224afde 21747075 PMC3140073

[2] Golombeck KS , Bonte K , Monig C , et al. Evidence of a pathogenic role for CD8^+^ T cells in anti‐GABA_B_ receptor limbic encephalitis. Neurology—Neuroimmunology Neuroinflammation. 2016;3(3):e232. 10.1212/nxi.0000000000000232 PMC4853055 27213174

[3] Ramanathan S , Mohammad SS , Brilot F , Dale RC . Autoimmune encephalitis: recent updates and emerging challenges. J Clin Neurosci. 2014;21(5):722‐730. 10.1016/j.jocn.2013.07.017 24246947

[4] Guan HZ , Ren HT , Cui LY . Autoimmune encephalitis: an expanding frontier of neuroimmunology. Chin Med J (Engl). 2016;129(9):1122‐1127. 10.4103/0366-6999.180514 27098800 PMC4852682

[5] Jarius S , Steinmeyer F , Knobel A , et al. GABA_B_ receptor antibodies in paraneoplastic cerebellar ataxia. J Neuroimmunol. 2013;256(1):94‐96. 10.1016/j.jneuroim.2012.12.006 23332614

[6] Quek AML , O'Toole O . Autoimmune epilepsy: the evolving science of neural autoimmunity and its impact on epilepsy management. Semin Neurol. 2018;38(3):290‐302. 30011410 10.1055/s-0038-1660860

[7] Maureille A , Fenouil T , Joubert B , et al. Isolated seizures are a common early feature of paraneoplastic anti‐GABA_B_ receptor encephalitis. J Neurol. 2019;266(1):195‐206. 10.1007/s00415-018-9132-0 30460450

[8] Zeng W , Cao L , Zheng J , Yu L . Clinical characteristics and long‐term follow‐up of seven cases of anti‐GABABR encephalitis in patients of Han Chinese descent. Neurol Sci. 2020;41(2):373‐378. 10.1007/s10072-019-04095-9 31659584 PMC7005084

[9] Chung HY , Wickel J , Voss A , et al. Autoimmune encephalitis with anti‐IgLON5 and anti‐GABA_B_‐receptor antibodies: A case report. Medicine (Baltimore). 2019;98(20):e15706. 10.1097/MD.0000000000015706 31096519 PMC6531245

[10] Zhang W , Wang X , Shao N , Ma R , Meng H . Seizure characteristics, treatment, and outcome in autoimmune synaptic encephalitis: a long‐term study. Epilepsy Behav. 2019;94:198‐203. 10.1016/j.yebeh.2018.10.038 30974347

[11] Gresa‐Arribas N , Titulaer MJ , Torrents A , et al. Antibody titres at diagnosis and during follow‐up of anti‐NMDA receptor encephalitis: a retrospective study. Lancet Neurol. 2014;13(2):167‐177. 10.1016/S1474-4422(13)70282-5 24360484 PMC4006368

[12] Joubert B , Honnorat J . Autoimmune channelopathies in paraneoplastic neurological syndromes. Biochim Biophys Acta. 2015;1848(10, Part B):2665‐2676. 10.1016/j.bbamem.2015.04.003 25883091

[13] Dogan Onugoren M , Deuretzbacher D , Haensch CA , et al. Limbic encephalitis due to GABA_B_ and AMPA receptor antibodies: a case series. J Neurol Neurosurg Psychiatry. 2015;86(9):965‐972. 10.1136/jnnp-2014-308814 25300449

[14] Kelley BP , Patel SC , Marin HL , Corrigan JJ , Mitsias PD , Griffith B . Autoimmune encephalitis: pathophysiology and imaging review of an overlooked diagnosis. Am J Neuroradiol. 2017;38(6):1070‐1078. 10.3174/ajnr.A5086 28183838 PMC7960083

[15] Cui J , Bu H , He J , et al. The gamma‐aminobutyric acid‐B receptor (GABA_B_) encephalitis: clinical manifestations and response to immunotherapy. Int J Neurosci. 2018;128(7):627‐633. 10.1080/00207454.2017.1408618 29166136

[16] Shen K , Xu Y , Guan H , et al. Paraneoplastic limbic encephalitis associated with lung cancer. Sci Rep. 2018;8(1):6792. 10.1038/s41598-018-25294-y 29717222 PMC5931551

[17] Bettler B , Kaupmann K , Mosbacher J , Gassmann M . Molecular structure and physiological functions of GABA_B_ receptors. Physiol Rev. 2004;84(3):835‐867. 10.1152/physrev.00036.2003 15269338

[18] Spatola M , Petit‐Pedrol M , Simabukuro MM , et al. Investigations in GABA_A_ receptor antibody‐associated encephalitis. Neurology. 2017;88(11):1012‐1020. 10.1212/wnl.0000000000003713 28202703 PMC5384834

[19] Lancaster E , Lai M , Peng X , et al. Antibodies to the GABA_B_ receptor in limbic encephalitis with seizures: case series and characterisation of the antigen. Lancet Neurol. 2010;9(1):67‐76. 10.1016/S1474-4422(09)70324-2 19962348 PMC2824142

[20] Kim TJ , Lee ST , Shin JW , et al. Clinical manifestations and outcomes of the treatment of patients with GABA_B_ encephalitis. J Neuroimmunol. 2014;270(1):45‐50. 10.1016/j.jneuroim.2014.02.011 24662003

[21] Qiao S , Zhang YX , Zhang BJ , et al. Clinical, imaging, and follow‐up observations of patients with anti‐GABA_B_ receptor encephalitis. Int J Neurosci. 2017;127(5):379‐385. 10.1080/00207454.2016.1176922 27066808

[22] Höftberger R , Titulaer MJ , Sabater L , et al. Encephalitis and GABAB receptor antibodies: novel findings in a new case series of 20 patients. Neurology. 2013;81(17):1500‐1506. 10.1212/WNL.0b013e3182a9585f 24068784 PMC3888170

[23] Guan HZ , Ren HT , Yang XZ , et al. Limbic encephalitis associated with anti‐γ‐aminobutyric acid B receptor antibodies: a case series from China. Chin Med J (Engl). 2015;128(22):3023‐3028. 10.4103/0366-6999.168989 26608981 PMC4795264

[24] Heine J , Prüss H , Bartsch T , Ploner CJ , Paul F , Finke C . Imaging of autoimmune encephalitis—relevance for clinical practice and hippocampal function. Neuroscience. 2015;309:68‐83. 10.1016/j.neuroscience.2015.05.037 26012492

